# Supplementary material for: Circulating cell-free RNA in blood as a host response biomarker for detection of tuberculosis
Source: Nat Commun. 2024 Jun 10;15:4949. doi: 10.1038/s41467-024-49245-6 (PMC11164910; doi:10.1038/s41467-024-49245-6)
Supplement: Supplementary file 1 — Supplementary Information [file 41467_2024_49245_MOESM1_ESM.pdf]

Supplementary Information for:
Circulating cell-free RNA in blood as a host response biomarker for detection of tuberculosis

Authors: Adrienne Chang[1,#], Conor J. Loy[1#], Daniel Eweis-LaBolle[1#], Joan S. Lenz[1], Amy Steadman[2], Alfred Andama[3], Nguyen Viet Nhung[4], Charles Yu[5], William Worodria[3], Claudia M. Denkinger[6], Payam Nahid[7], Adithya Cattamanchi[7,8], Iwijn De Vlaminck[1,*]

Affiliations: [1]Meinig School of Biomedical Engineering, Cornell University, Ithaca, NY, USA; [2]Global Health Labs, Inc. Bellevue, WA; [3]Walimu, Kampala, Uganda; [4]National Lung Hospital, Hanoi, Vietnam; [5]De La Salle Medical and Health Sciences Institute, Dasmarinas, Philippines; [6]University Hospital Heidelberg & German Center of Infection Research, Heidelberg, Germany; [7]Center for Tuberculosis, University of California San Francisco, San Francisco, CA, USA; [8]Division of Pulmonary and Critical Care Medicine, University of California Irvine, Orange, CA, USA

# These authors contributed equally
*Corresponding author at: vlaminck@cornell.edu

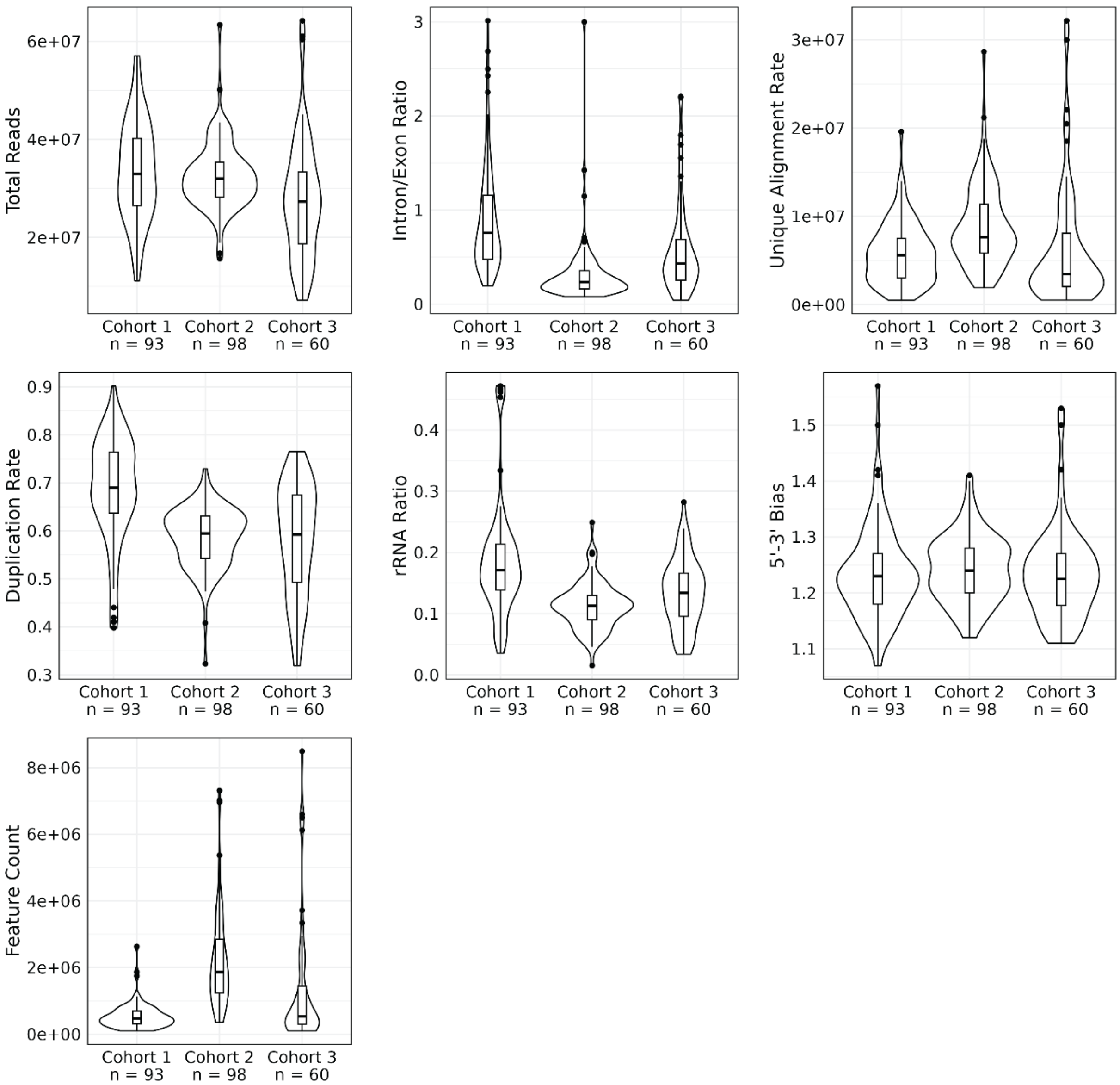

Supplementary Figure 1. Quality control metrics used to filter samples. Samples were filtered on the basis of DNA contamination (estimated by the intron/exon ratio), total counts (calculated using feature counts), rRNA contamination, and RNA degradation (estimated by the 5’-3’ bias).

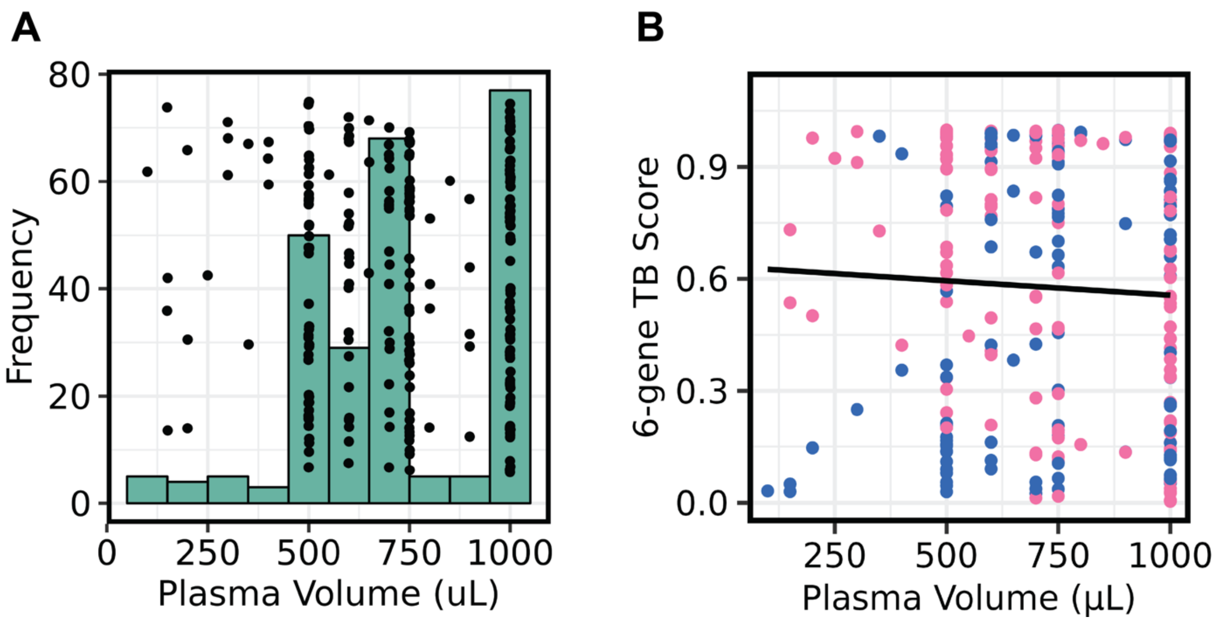

Supplementary Figure 2. No relation between plasma volume and signature score. A) Histogram showing the distribution of plasma sample volumes used in this study (n = 251)**. B)** Lack of correlation of the 6-gene TB score with plasma sample volume (Pearson correlation r = -0.036, p = 0.57). Color indicates disease status (pink = TB positive; blue = TB negative, n = 251).

Model	Training ROC-AUC [95% CI]	Test ROC-AUC [95% CI]	Training Accuracy [95% CI]	Test Accuracy [95% CI]	Training Sensitivity [95% CI]	Test Sensitivity [95% CI]	Training Specificity [95% CI]	Test Specificity [95% CI]
**C5**	1 [1-1]	0.93 [0.87-0.99]	1 [1-1]	0.87 [0.78-0.95]	1 [1-1]	0.91 [0.8-1.02]	1 [1-1]	0.81 [0.69-0.94]
**EXTRATREES**	1 [1-1]	0.93 [0.88-0.99]	1 [1-1]	0.8 [0.7-0.9]	1 [1-1]	0.88 [0.75-1.01]	1 [1-1]	0.7 [0.56-0.85]
**GLM**	0.58 [0.49-0.67]	0.57 [0.44-0.7]	0.39 [0.31-0.48]	0.43 [0.3-0.55]	0.34 [0.23-0.45]	0.47 [0.29-0.66]	0.46 [0.33-0.59]	0.37 [0.21-0.54]
**GLMNETLasso**	1 [0.99-1]	0.89 [0.81-0.97]	0.98 [0.96-1.01]	0.77 [0.66-0.88]	0.97 [0.93-1.01]	0.79 [0.64-0.95]	1 [1-1]	0.74 [0.59-0.89]
**GLMNETRidge**	0.91 [0.85-0.96]	0.92 [0.86-0.99]	0.86 [0.8-0.92]	0.82 [0.72-0.92]	0.9 [0.82-0.98]	0.85 [0.72-0.99]	0.81 [0.72-0.9]	0.78 [0.64-0.92]
**KNN**	0.88 [0.82-0.94]	0.9 [0.83-0.98]	0.84 [0.78-0.9]	0.79 [0.68-0.89]	0.89 [0.81-0.98]	0.85 [0.71-0.99]	0.77 [0.68-0.87]	0.7 [0.56-0.85]
**LDA**	1 [1-1]	0.59 [0.45-0.74]	1 [1-1]	0.54 [0.42-0.67]	1 [1-1]	0.56 [0.38-0.74]	1 [1-1]	0.52 [0.35-0.69]
**NB**	0.95 [0.92-0.99]	0.89 [0.81-0.97]	0.9 [0.85-0.95]	0.82 [0.72-0.92]	0.92 [0.85-0.99]	0.82 [0.68-0.96]	0.88 [0.8-0.95]	0.81 [0.68-0.95]
**NNET**	0.9 [0.85-0.96]	0.92 [0.86-0.99]	0.86 [0.8-0.92]	0.82 [0.72-0.92]	0.9 [0.82-0.98]	0.85 [0.72-0.99]	0.81 [0.72-0.9]	0.78 [0.64-0.92]
**PAM**	0.89 [0.83-0.95]	0.91 [0.84-0.98]	0.85 [0.79-0.91]	0.75 [0.65-0.86]	0.92 [0.84-0.99]	0.85 [0.7-1]	0.77 [0.68-0.86]	0.63 [0.48-0.78]
**RF**	1 [1-1]	0.94 [0.88-0.99]	1 [1-1]	0.87 [0.78-0.95]	1 [1-1]	0.88 [0.76-1]	1 [1-1]	0.85 [0.73-0.97]
**RPART**	0.83 [0.76-0.9]	0.84 [0.75-0.93]	0.83 [0.77-0.9]	0.85 [0.76-0.94]	0.84 [0.74-0.93]	0.94 [0.84-1.04]	0.82 [0.74-0.91]	0.74 [0.6-0.88]
**SVMLIN**	0.93 [0.87-0.98]	0.93 [0.87-0.99]	0.88 [0.82-0.93]	0.82 [0.72-0.92]	0.92 [0.84-0.99]	0.91 [0.79-1.03]	0.82 [0.74-0.91]	0.7 [0.56-0.85]
**SVMRAD**	0.97 [0.94-0.99]	0.91 [0.84-0.98]	0.92 [0.88-0.97]	0.85 [0.76-0.94]	0.89 [0.81-0.97]	0.82 [0.69-0.96]	0.96 [0.92-1.01]	0.89 [0.78-1]

Supplementary Table 1. Performance metrics of the 15 models on the training and test sets (n = 130 for all training set intervals, n = 61 for all test set intervals).

Step #	ENSEMBL ID	Gene Name	ROC-AUC [95% CI]	Sensitivity [95% CI]	Specificity [95% CI]
1	ENSG00000154451.14	*GBP5*	0.873 [0.811-0.936]	0.836 [0.751-0.921]	0.825 [0.726-0.923]
2	ENSG00000104765.16	*BNIP3L*	0.88 [0.812-0.948]	0.973 [0.935 – 1]	0.737 [0.623-0.851]
3	ENSG00000067082.15	*KLF6*	0.9 [0.844-0.957]	0.918 [0.855-0.981]	0.842 [0.747-0.937]
4	ENSG00000135636.15	*DYSF*	0.912 [0.859-0.965]	0.89 [0.819-0.962]	0.877 [0.792-0.962]
5	ENSG00000002834.18	*LASP1*	0.915 [0.864-0.967]	0.918 [0.855-0.981]	0.877 [0.792-0.962]
6	ENSG00000169564.7	*PCBP1*	0.921 [0.872-0.971]	0.945 [0.893-0.997]	0.877 [0.792-0.962]

Supplementary Table 2. Genes and model performance of each step in the greedy forward search in the training dataset (n = 130 for all intervals calculated here).

Gene	Semiquantitative Xpert Ultra result	Group 1	Group 2	Group 1 Mean	Group 2 Mean	Adjusted p-value
GBP5	Negative	END TB	R2D2	7.07	6.63	0.00772
		END TB	TBSQ	7.07	6.3	0.000334
		R2D2	TBSQ	6.63	6.3	0.0391
	Low	END TB	R2D2	8.2	7.83	0.399
		END TB	TBSQ	8.2	7.5	0.326
		R2D2	TBSQ	7.83	7.5	0.4
	Medium	END TB	R2D2	8.37	7.73	0.0446
		END TB	TBSQ	8.37	7.83	0.246
		R2D2	TBSQ	7.73	7.83	0.766
	High	END TB	R2D2	8.64	8.23	0.108
		END TB	TBSQ	8.64	7.9	0.0227
		R2D2	TBSQ	8.23	7.9	0.117

Supplementary Table 3. Comparison of GBP5 normalized counts (VST) by cohort and semiquantitative Xpert Ultra result, two-sided Wilcoxon test.

Group 1	Group 2	Group 1 Mean	Group 2 Mean	Adjusted p-value
Negative	Low	0.303	0.757	$8.89x10^{-11}$
Negative	Medium	0.303	0.81	$1.95x10^{-16}$
Negative	High	0.303	0.892	$6.37x10^{-21}$
Low	Medium	0.757	0.81	0.618
Low	High	0.757	0.892	0.0728
Medium	High	0.81	0.892	0.0957

Supplementary Table 4. Comparison of 6-gene TB score and semiquantitative Xpert Ultra results for all samples, two-sided Wilcoxon test.

Semiquantitative Xpert Ultra result	Group 1	Group 2	Group 1 Mean	Group 2 Mean	Adjusted p-value
Negative	END TB	R2D2	0.305	0.258	0.735
	END TB	TB2	0.305	0.386	0.34
	R2D2	TB2	0.258	0.386	0.168
Low	END TB	R2D2	0.736	0.751	0.811
	END TB	TB2	0.736	0.803	0.811
	R2D2	TB2	0.751	0.803	0.811
Medium	END TB	R2D2	0.785	0.783	0.622
	END TB	TB2	0.785	0.879	0.614
	R2D2	TB2	0.783	0.879	0.622
**High**	**END TB**	**R2D2**	**0.883**	**0.877**	**0.925**
	**END TB**	**TB2**	**0.883**	**0.928**	**0.925**
	**R2D2**	**TB2**	**0.877**	**0.928**	**0.925**

Supplementary Table 5. Comparison of 6-gene TB score and cohort, two-sided Wilcoxon test.

Gene	Group 1	Group 2	Base Mean	Log2 Fold Change	Adjusted p-value
MARCO	Plasma<br>TB Positive	Plasma<br>TB Negative	13.4	0.9	9.56×10^{-7}
	Whole Blood<br>Cohort 3<br>TB Positive	Whole Blood<br>Cohort 3<br>TB Negative	4.65	0.747	0.14
CASP4	Plasma<br>TB Positive	Plasma<br>TB Negative	107	0.498	8.73×10^{-16}
	Whole Blood<br>Cohort 3<br>TB Positive	Whole Blood<br>Cohort 3<br>TB Negative	294	0.796	9.21×10^{-7}
GBP1	Plasma<br>TB Positive	Plasma<br>TB Negative	148	1.23	6.55×10^{-23}
	Whole Blood<br>Cohort 3<br>TB Positive	Whole Blood<br>Cohort 3<br>TB Negative	407	1.47	3.85×10^{-5}
GBP2	Plasma<br>TB Positive	Plasma<br>TB Negative	204	0.816	1.71×10^{-18}
	Whole Blood<br>Cohort 3<br>TB Positive	Whole Blood<br>Cohort 3<br>TB Negative	547	1.07	1.55×10^{-8}
GBP4	Plasma<br>TB Positive	Plasma<br>TB Negative	69.8	0.777	3.78×10^{-13}
	Whole Blood<br>Cohort 3<br>TB Positive	TB2 (WB)<br>TB Negative	127	1.1	4.39×10^{-4}
GBP5	Plasma<br>TB Positive	Plasma<br>TB Negative	228	1.53	3.4×10^{-38}
	Whole Blood<br>Cohort 3<br>TB Positive	Whole Blood<br>Cohort 3<br>TB Negative	1,290	1.88	3.73×10^{-8}
STAT1	Plasma<br>TB Positive	Plasma<br>TB Negative	240	0.804	2.13×10^{-17}
	Whole Blood<br>Cohort 3<br>TB Positive	Whole Blood<br>Cohort 3<br>TB Negative	593	0.819	6.21×10^{-4}

Supplementary Table 6. DESeq2 comparisons of lung, pyroptosis, and necrosis-specific gene abundances between TB status for both plasma and whole blood. Positive log2 fold change values indicate elevated counts in the TB positive group. All comparisons were done using DESeq2 (two sided Wald test, Benjamini-Hochberg adjusted p-values).
