# Supplementary material for: Circulating cell-free RNA in blood as a host response biomarker for detection of tuberculosis
Source: Nat Commun. 2024 Jun 10;15:4949. doi: 10.1038/s41467-024-49245-6 (PMC11164910; doi:10.1038/s41467-024-49245-6)
Supplement: Supplementary file 3 — Description of Additional Supplementary Files [file 41467_2024_49245_MOESM3_ESM.pdf]

Description of Additional Supplementary Files

File Name: Supplementary Data 1 (SuppData1.tsv)
Description: Plasma cfRNA Differential abundances between TB positive and TB negative groups.

File Name: Supplementary Data 2 (SuppData2.txt)
Description: Plasma cfRNA IPA pathways of enriched genes and molecules in TB Positive versus TB negative groups.

File Name: Supplementary Data 3 (SuppData3.xslx)
Description: Plasma cfRNA KEGG, GO, and Hallmark pathways of enriched genes and molecules in TB Positive versus TB negative groups.

File Name: Supplementary Data 4 (SuppData4.tsv)
Description: Gene signature panels identified in whole blood RNA and plasma cfRNA to diagnose active TB.

File Name: Supplementary Data 5 (SuppData5.tsv)
Description: Whole Blood RNA Differential abundances between TB positive and TB negative groups.
