# Supplementary material for: Circulating cell-free RNA in blood as a host response biomarker for detection of tuberculosis
Source: Nat Commun. 2024 Jun 10;15:4949. doi: 10.1038/s41467-024-49245-6 (PMC11164910; doi:10.1038/s41467-024-49245-6)
Supplement: Supplementary file 9 — Reporting Summary [file 41467_2024_49245_MOESM9_ESM.pdf]

Corresponding author(s): Iwijn De Vlaminck
Last updated by author(s): Apr 10, 2024

Reporting Summary

Nature Portfolio wishes to improve the reproducibility of the work that we publish. This form provides structure for consistency and transparency in reporting. For further information on Nature Portfolio policies, see our Editorial Policies and the Editorial Policy Checklist.

Statistics

For all statistical analyses, confirm that the following items are present in the figure legend, table legend, main text, or Methods section.

n/a	Confirmed	
☐	☒	The exact sample size (*n*) for each experimental group/condition, given as a discrete number and unit of measurement
☐	☒	A statement on whether measurements were taken from distinct samples or whether the same sample was measured repeatedly
☐	☒	The statistical test(s) used AND whether they are one- or two-sided<br>*Only common tests should be described solely by name; describe more complex techniques in the Methods section.*
☐	☒	A description of all covariates tested
☐	☒	A description of any assumptions or corrections, such as tests of normality and adjustment for multiple comparisons
☐	☒	A full description of the statistical parameters including central tendency (e.g. means) or other basic estimates (e.g. regression coefficient) AND variation (e.g. standard deviation) or associated estimates of uncertainty (e.g. confidence intervals)
☐	☒	For null hypothesis testing, the test statistic (e.g. *F*, *t*, *r*) with confidence intervals, effect sizes, degrees of freedom and *P* value noted<br>*Give P values as exact values whenever suitable.*
☐	☒	For Bayesian analysis, information on the choice of priors and Markov chain Monte Carlo settings
☒	☐	For hierarchical and complex designs, identification of the appropriate level for tests and full reporting of outcomes
☐	☒	Estimates of effect sizes (e.g. Cohen's *d*, Pearson's *r*), indicating how they were calculated

Our web collection on statistics for biologists contains articles on many of the points above.

Software and code

Policy information about availability of computer code

Data collection	We did not use any software for data collection.
Data analysis	All data analysis scripts for this study have been made available on GitHub (https://github.com/DanielEweisLaBolle/cfRNA_TB). The following list contains the packages and software used and their version numbers:<br>Seqtk (v1.2), BBDuk (v38.90), STAR (v2.7.0f), featureCounts (v2.0.0), Picard MarkDuplicates (v2.19.2), SAMtools (v1.14), Qualimap (v2.2.1), BayesPrism (v1.1), ScanPy (v1.8.1), DESeq2 (v1.34.0), pheatmap (v1.0.12), QIAGEN Ingenuity Pathway Analysis software (v73620684), R (v4.1.3), Care (v6.0.90), pROC (v1.18.0).

For manuscripts utilizing custom algorithms or software that are central to the research but not yet described in published literature, software must be made available to editors and reviewers. We strongly encourage code deposition in a community repository (e.g. GitHub). See the Nature Portfolio guidelines for submitting code & software for further information.

Data

Policy information about availability of data

All manuscripts must include a data availability statement. This statement should provide the following information, where applicable:

- Accession codes, unique identifiers, or web links for publicly available datasets
- A description of any restrictions on data availability
- For clinical datasets or third party data, please ensure that the statement adheres to our policy

The raw sequencing data and de-identified RNA-seq count matrices generated in this study have been deposited in the Gene Expression Omnibus under the accession codes GSE255071, GSE255073, GSE255074. For alignment we used the Gencode GRCh38 human reference genome (v38, primary assembly). For cell type deconvolution via BayesPrism we used the Tabula Sapiens single-cell RNA-seq atlas10 (Release 1) as a reference.

Research involving human participants, their data, or biological material

Policy information about studies with human participants or human data. See also policy information about sex, gender (identity/presentation), and sexual orientation and race, ethnicity and racism.

Reporting on sex and gender

Males and Females were evenly distributed amongst cohorts and evenly split across Train, Test and Validation sets.

Reporting on race, ethnicity, or other socially relevant groupings

We provide country-based analysis in Figure 3F to analyze the effect of the country of origin in classifying patients as TB positive or negative.

Population characteristics

Cohort 1 (Formerly END TB)
93 plasma samples were collected from individuals with a cough of at least two weeks seeking tuberculosis treatment in Uganda. This cohort consisted of 35 females and 58 males.
Cohort 2 (Formerly R2D2)
98 plasma samples were collected from individuals with a cough of at least two weeks seeking tuberculosis treatment in Uganda, the Philippines, or Vietnam. This cohort consisted of 41 females and 57 males.
Cohort 3 (Validation Cohort and Whole Blood samples)
60 plasma samples and 60 whole blood samples were collected from individuals with a cough of at least two weeks seeking tuberculosis treatment in Uganda. This cohort consisted of 19 females and 41 males.

Recruitment

We performed a case-control study. We analyzed plasma samples from a total of 251 individuals with a cough lasting at least two weeks who were enrolled in three different cohorts (Cohort 1, Cohort 2, and Cohort 3) at outpatient clinics in Uganda, Vietnam, and the Philippines (Table 1 and Figure 1A). Individuals included in the "TB positive" group were required to have 1) a positive Xpert MTB/RIF Ultra on sputum, urine, or contaminated Mycobacterial Growth Indicator Tube (MGIT) specimen; 2) a positive sputum MGIT or solid culture; or, 3) two trace Xpert Ultra results on sputum or contaminated MGIT. All other individuals ("TB negative" group) had at least one negative Xpert Ultra result, two negative cultures in MGIT or solid media and repeat negative sputum tests and/or clinical improvement without TB treatment at two to three month follow-up.

Ethics oversight

The protocols for this study were approved locally at each site by Institutional Review Boards: Cornell University (protocols IRB0145569, 1902008555); UCSF IRB 20-32670 (protocol 20-32670); University of Heidelberg Ethics Committee of the Medical Faculty (S-539/2020); the Makerere University, College of Health Sciences, School of Medicine, Research Ethics Committee 2020-182 (protocol 2017-020); Vietnam National Lung Hospital Ethical Committee for Biological Medical Research: 566/2020/NCKH (protocol 566/2020/NCKH), and De La Salle Health Sciences Institute Independent Ethics Committee 2020-33-02-A (protocol 2020-33-02-A).

Note that full information on the approval of the study protocol must also be provided in the manuscript.

Field-specific reporting

Please select the one below that is the best fit for your research. If you are not sure, read the appropriate sections before making your selection.

☒ Life sciences ☐ Behavioural & social sciences ☐ Ecological, evolutionary & environmental sciences

For a reference copy of the document with all sections, see nature.com/documents/nr-reporting-summary-flat.pdf

Life sciences study design

All studies must disclose on these points even when the disclosure is negative.

Sample size

We collected as many patient samples as possible that fit the criteria in each cohort (n=251). A detailed description of the sample sizes in each cohort is given in Table 1 and Figure 1A.

Data exclusions

No data was excluded from the analysis apart from samples that did not meet QC metrics as described in the Methods section.

Replication	Given the rarity and low volumes of samples, we did not have enough material to replicate individual measurements. Reproducibility was tested by obtaining samples from individuals enrolled in three distinct cohorts.
Randomization	Sample splitting for the training and test sets and the greedy forward search analysis was randomized using the set.seed function in R. Samples were split accounting for TB status, Cohort, and HIV status. The seed was set to 87 (available in the code provided on github).
Blinding	Investigators were not blinded to group allocation because this information was available from prior studies by the same investigators.

Reporting for specific materials, systems and methods

We require information from authors about some types of materials, experimental systems and methods used in many studies. Here, indicate whether each material, system or method listed is relevant to your study. If you are not sure if a list item applies to your research, read the appropriate section before selecting a response.

Materials & experimental systems

n/a	Involved in the study
☒	☐ Antibodies
☒	☐ Eukaryotic cell lines
☒	☐ Palaeontology and archaeology
☒	☐ Animals and other organisms
☒	☐ Clinical data
☒	☐ Dual use research of concern
☒	☐ Plants

Methods

n/a	Involved in the study
☒	☐ ChIP-seq
☒	☐ Flow cytometry
☒	☐ MRI-based neuroimaging

Plants

Seed stocks	*Report on the source of all seed stocks or other plant material used. If applicable, state the seed stock centre and catalogue number. If plant specimens were collected from the field, describe the collection location, date and sampling procedures.*
Novel plant genotypes	*Describe the methods by which all novel plant genotypes were produced. This includes those generated by transgenic approaches, gene editing, chemical/radiation-based mutagenesis and hybridization. For transgenic lines, describe the transformation method, the number of independent lines analyzed and the generation upon which experiments were performed. For gene-edited lines, describe the editor used, the endogenous sequence targeted for editing, the targeting guide RNA sequence (if applicable) and how the editor was applied.*
Authentication	*Describe any authentication procedures for each seed stock used or novel genotype generated. Describe any experiments used to assess the effect of a mutation and, where applicable, how potential secondary effects (e.g. second site T-DNA insertions, mosiacism, off-target gene editing) were examined.*
